# Supplementary material for: The histone deacetylase complex MiDAC regulates a neurodevelopmental gene expression program to control neurite outgrowth
Source: eLife. 2020 Apr 16;9:e57519. doi: 10.7554/eLife.57519 (PMC7192582; doi:10.7554/eLife.57519)
Supplement: Supplementary file 1. [file elife-57519-supp1.docx]

**Supplementary file 1.** List of guide RNA and deep sequencing primer sequences used for CRISPR/Cas9-mediated genome editing of *Dnttip1* and *Elmsan1* in mESCs.

| **gRNAs** | **Sequence (5’ to 3’)** |
| --- | --- |
| *Dnttip1* gRNA | ACAUCGGCAGGUGCAGCGAA |
| *Elmsan1* gRNA | GCUCUCUUGGCGGCUAAGGC |
|  |  |
| **Deep Sequencing Primers** | **Sequence (5’ to 3’)** |
| Dnttip1.NGS.F  partial Illumina adaptors (upper case) | CACTCTTTCCCTACACGACGCTCTTCCGATCTccaaggtgggagaagtgagaagcagg |
| Dnttip1.NGS.R  partial Illumina adaptors (upper case) | GTGACTGGAGTTCAGACGTGTGCTCTTCCGATCTccccactcctgtgctctttacgggc |
| Elmsan1.NGS.F  partial Illumina adaptors (upper case) | CACTCTTTCCCTACACGACGCTCTTCCGATCTaaacaggggctcaactcctgaacct |
| Elmsan1.NGS.R  partial Illumina adaptors (upper case) | GTGACTGGAGTTCAGACGTGTGCTCTTCCGATCTcttgtgctcggtggggtactgctca |
