# Supplementary material for: The histone deacetylase complex MiDAC regulates a neurodevelopmental gene expression program to control neurite outgrowth
Source: eLife. 2020 Apr 16;9:e57519. doi: 10.7554/eLife.57519 (PMC7192582; doi:10.7554/eLife.57519)
Supplement: Supplementary file 5. [file elife-57519-supp5.docx]

**Supplementary file 5.** List of primer sequences used for qRT-PCR analysis.

| **Gene** | **Forward Primer (5’ to 3’)** | **Reverse Primer (5’ to 3’)** |
| --- | --- | --- |
| *Dnttip1* | CCCATGAGCTTCCAGGAATA | CGAGATCCCAACACAAAGGT |
| *Elmsan1* | AAAGGATGGCAGTGGTTCTG | CCTCAGTTGGGTCCACAGTT |
| *Hdac1* | GGAAATCTATCGCCCTCACA | AACAGGCCATCGAATACTGG |
| *Hdac2* | CGGAGAAGATTGTCCGGTGT | ATTTCACAGCCCCAGCAACT |
| *Oct4* | ATGGCATACTGTGGACCTCA | AGCAGCTTGGCAAACTGTTC |
| *Nanog* | CTCATCAATGCCTGCAGTTTTTCA | CTCCTCAGGGCCCTTGTCAGC |
| *Sox2* | GGTTACCTCTTCCTCCCACTCCAG | TCACATGTGCGACAGGGGCAG |
| *Pax6* | TGGTATTCTCTCCCCCTCCT | TAAGGATGTTGAACGGGCAG |
| *Nes* | TGAGGGTCAGGTGGTTCTG | AGAGCAGGGAGGGACATTC |
| *Tubb3* | TCCGAGTACCAGCAGTACCA | GGCTTCCGATTCCTCGTCAT |
| *Map2* | CCCCATGGCAAACCGCTAA | AAGGCAAGTAGGAGAGGGAGA |
| *Slit3* | AGTTGTCTGCCTTCCGACAG | GCACTCGGAGGGATCTTAGC |
| *Ntn1* | CACTGCCACTACTGCAAGGA | TAGCCTTTGGCACATCGGTT |
| *Ncam1* | AGAGGACGGGAACTCCATCA | GGTTTCCACTCAGAGGCGAG |
| *Robo3* | GAATCGCCGAGAGGAACCAA | CACATCGGTTGACCAGGGAA |
| *Unc5b* | GTGCTGACCCTGGAGACATC | GAGAACCGCTACCACCACAA |
| *Spry4* | TTCGGGGATTTACACAGACG | CTGCTGTCAAGGAGGGGC |
| *Id1* | GAGGCGGCATGTGTTCCA | GAAGGGCTGGAGTCCATCTG |
| *Pacsin1* | GCAGGACCACTTCTCTCACC | GCTTGTAGTTCCCCACCTCC |
| *Gapdh* | TGTGTCCGTCGTGGATCTGA | CCTGCTTCACCACCTTCTTGA |
