# Supplementary material for: The histone deacetylase complex MiDAC regulates a neurodevelopmental gene expression program to control neurite outgrowth
Source: eLife. 2020 Apr 16;9:e57519. doi: 10.7554/eLife.57519 (PMC7192582; doi:10.7554/eLife.57519)
Supplement: Supplementary file 6. [file elife-57519-supp6.docx]

**Supplementary file 6.** List of primer sequences used for ChIP qPCR analysis.

| **Gene** | **Target Type** | **Forward Primer (5’ to 3’)** | **Reverse Primer (5’ to 3’)** |
| --- | --- | --- | --- |
| *Slit3* | Promoter (p1) | CACTGTGGTGAGGAGAGCTG | CTGAACACCCATCCAGCCAT |
| *Slit3* | Enhancer 1 (e1) | CCTGCACTCCCTCACTCTTC | GCTGAACAATCATCCCAGGT |
| *Slit3* | Enhancer 2 (e2) | TAAGCTTGCTTGGTGCCTTT | TGTTGCCCTTGGTAACCTTC |
| *Slit3* | Intragenic Control Region (NC) | TCTTGTTCAGAATGCATTTACAGAT | TCTGTATGTCTCTCAGCAACCT |
| *Ntn1* | Promoter 1 (p1) | TCAAACTGGGGGCTATGAAG | AGCTCCTGGCCAAACACTAA |
| *Ntn1* | Promoter 2 (p2) | TATGCCCATTCTCCTTGCTC | GGCCAGGCAAACTTTTCTTT |
| *Ntn1* | Enhancer (e1) | TTTGAGGCAGGGTCTCAGTT | TGAGGGAGGTGGAGTCAAAC |
| *Spry4* | Promoter (p1) | TCAGAAATTCGCTTGCCTCT | TGGCCAACGCTATTTAAACC |
| *Spry4* | Enhancer 1 (e1) | ACGCATTGTTGCTTCTGTTG | TCATGGCTTGGGACCATATT |
| *Spry4* | Enhancer 2 (e2) | GGAGGAAGGAGAAAGGGATG | CCACCAATCACCACAGACAG |
| *Spry4* | Intragenic Control Region (NC) | TCACCAGCAGGGCAATTTTATG | AGGAGCCAAAAATCAGCACCA |
| *Id1* | Promoter (p1) | CACCTGGGGTCAGAACATCT | CTGTTGGAAGATTGGGCTGT |
| *Id1* | Enhancer 1 (e1) | CTGGGAATGTAGGCACCTGT | CTTAGCTGTTGCCCTGGAAG |
| *Id1* | Enhancer 2 (e2) | TCCTGCAGCATGTAATCGAC | ATACTCTGCCTCGGACCTCA |
| *Robo3* | Promoter | GGGCTAAGGACAATACTGTAGGA | GGCTCATCAACCCTGGAATGT |
| *Unc5b* | Promoter | CTCATTTCTCAAATGGGGCAGT | GCCAGCACTGGGATGACTTA |
